# P-956. Perception of the Fellows/Faculty on Inpatient Expectations and Academic Half-Day

**DOI:** 10.1093/ofid/ofae631.1146

**Published:** 2025-01-29

**Authors:** Georgina Osorio, Geraldine Vargas, Stanley Yancovitz

**Affiliations:** Mount Sinai Health System, Icahn School of Medicine, New York, New York; Mount Sinai Health System, Icahn School of Medicine, New York, New York; Mount Sinai Health System, Icahn School of Medicine, New York, New York

## Abstract

**Background:**

Increased volume of Infectious Diseases (ID) inpatient consults has spear-headed training programs to launch efforts to mitigate burnout for optimal quality patient care and physician well-being including well-structured interventions such as academic half-day (AHD) to protect education time for fellows. This project describes inpatient strategies, electronic medical record (EMR) utilization, and perspectives on implementation of AHD by ID inpatient faculty and fellows at a large academic institution.

**Methods:**

An anonymous survey on daily inpatient schedules, EMR utilization, and AHD feedback was distributed to the ID Section at a NYC urban hospital system in 3/2024.

**Results:**

21 members of the ID section completed the survey (14 fac­ulty, 7 fellows). Daily inpatient schedules: When asked what time fellows should start workday, median response was 8AM-8:30 AM from both fellows (range, 8AM-9AM) and faculty (range, 7AM–9AM). When asked what time fellows should end work day, median response was 4:30PM-5PM from fellows (range, 4:30PM-5:30PM) and 5:30PM-6PM from faculty (range, 4:30PM-6:30PM). Fellows reported starting workday 7:30AM-8AM (range, 7AM-8:30AM) and ending 5:30PM-6PM (range, 4:30PM-6:30PM), while faculty reported starting 8:30AM-9AM (range, 6AM-9AM) and ending 6PM-6:30PM (range, 4:30PM-7PM). EMR utilization: fellows reported taking 4-6 hours to write notes (range, 2-6) and “about half the time” changing secure chat to “offline” when not on call versus faculty reported taking 2-4 hours (range, 1-6) to write notes and “never” setting EPIC secure chat to “offline.” AHD: Both groups reported AHD advantages were protected time for education, ability for fellows to focus on continuity clinic, in-person, and amplification of teamwork/mentorship. Fellows reported no disadvantages with AHD while faculty noted concerns of lost of continuity of care and not preparing fellows for clinical expectations as they transition to faculty.

**Conclusion:**

Both fellows and faculty report different strategies with EMR utilization. AHD feedback was overall positive by both groups, but faculty expressed concerns regarding impact to continuity of care. AHD evaluation and strategies to mitigate burnout are ongoing including leveraging EMR.

**Disclosures:**

**All Authors**: No reported disclosures

